# Bayesian Regression Facilitates Quantitative Modeling of Cell Metabolism

**DOI:** 10.1021/acssynbio.3c00662

**Published:** 2024-04-05

**Authors:** Teddy Groves, Nicholas Luke Cowie, Lars Keld Nielsen

**Affiliations:** † The Novo Nordisk Foundation Center for Biosustainability, DTU, Kongens Lyngby 2800, Denmark; ‡ Australian Institute for Bioengineering and Nanotechnology (AIBN), The University of Queensland, St Lucia 4067, Australia

**Keywords:** Bayesian inference, kinetic models of cell metabolism, multiomics integration, ordinary differential equations, regulatory anlaysis

## Abstract

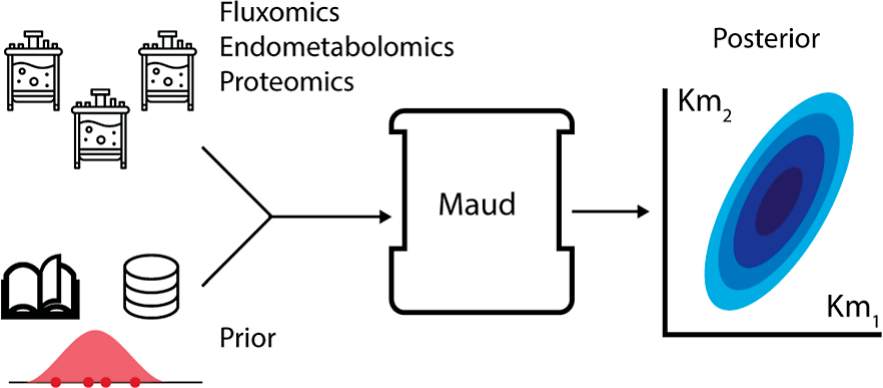

This paper presents Maud, a command-line application that implements Bayesian statistical inference for kinetic models of biochemical metabolic reaction networks. Maud takes into account quantitative information from omics experiments and background knowledge as well as structural information about kinetic mechanisms, regulatory interactions, and enzyme knockouts. Our paper reviews the existing options in this area, presents a case study illustrating how Maud can be used to analyze a metabolic network, and explains the biological, statistical, and computational design decisions underpinning Maud.

## 1 Introduction

A kinetic model of cellular metabolism aims to express what is known about a cellular process in the form of an in silico representation of the underlying network of chemical reactions. Kinetic models can be used to improve production of target molecules, determine regulatory networks,^1^ and identify potential drug targets.^2,3^ However, the use of kinetic models in practice is hindered by their dependence on noisy and uncertain information sources. Quantitative in vivo measurements of chemical abundances and in vitro measurements relating to kinetic parameters, both contain vital information but are notoriously inaccurate.^4−6^ Practically useful kinetic modeling therefore requires a principled statistical approach that encompasses multiple possible model parametrizations.

Bayesian statistical inference can combine the structural information implicit in kinetic models with knowledge about metabolic parameters and information from omics measurements.^7,8^ However, kinetic models pose serious computational challenges for Bayesian inference.^9,10^

The scope of a kinetic model is defined by a stoichiometric matrix, *S*, in which rows represent metabolites, columns represent reactions, and matrix elements *s*_*ij*_ represent the stoichiometric coefficient of metabolite *i* in reaction *j*. The change in metabolite concentrations is
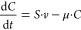
1where *C* represents a vector of metabolite concentrations, *v* is a vector of reaction rates, and μ is the growth rate. The second term represents dilution due to cell growth.

In a kinetic model, the rates, *v*, are expressed as a function of the enzyme concentrations, *E*, the metabolite concentrations, *C*, and a set of parameters θ
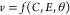
2

The parameters must include sufficient boundary concentrations and fluxes to solve.^1^

It is common to assume a pseudosteady state for metabolites, i.e., the rate of fluxes toward any metabolite is much greater than the rate of change in concentration, . Moreover, the dilution effect is assumed to be minimal, μ·*C* ≪ *v⃗* (true unless the concentration is very high). Finally, the enzyme concentration is assumed to be constant for the period considered and hence part of the parameters.

Given these assumptions and a set of values for θ, a set of steady-state metabolite concentrations and fluxes can be found by solving for *C* the following algebraic equation
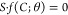
3

In a fermentation context,^3^ captures the rapid kinetics inside the cell, while another set of ODEs would be used to describe the external substrate and product concentrations, which could act as boundary parameters to.^3^

In the context of kinetic modeling, Bayesian inference is appealing because it allows uncertainty to be represented appropriately without sacrificing mechanistic accuracy. Measurement uncertainty can naturally be represented in a Bayesian measurement model, whereas the prior model can represent quantitative uncertainty about kinetic parameters. Finally, kinetic rate laws can be represented in Bayesian data generation models with arbitrarily high fidelity. See Gelman et al.^11^ for more details about Bayesian inference and Gelman et al.^12^ for a discussion of practical Bayesian workflow.

Another advantage is that Bayesian inference problems are well-posed, even when not all parameters are strongly identified. Sloppy parameter models in which measurable quantities are sensitive to combinations of parameters but not to individual marginal parameter values are ubiquitous in models of biological systems.^9,13^ The parameter correlation structure represents the set of potential models that describe the observed data. As we demonstrate later, capturing this correlation structure is difficult outside of a fully Bayesian context.

Previous Bayesian kinetic models have either sacrificed mechanistic accuracy or attempted to fit realistic kinetic models using obsolete or unreliable computational methods.

The most popular algorithm for fitting Bayesian statistical models is Markov Chain Monte Carlo (MCMC). Modern MCMC algorithms enable exploration of high-dimensional posterior distributions, have robust failure diagnostics,^14^ and can incorporate fast numerical solvers, thereby making inference feasible for Bayesian kinetic models. Nonetheless, the kinetic modeling literature reports an aversion to MCMC, rooted mainly in concerns about sampling time and the presumed difficulty of implementing the required statistical model.^7,15^ We are only aware of two recent attempts to implement a Bayesian kinetic modeling approach using MCMC. Stapor et al.^16^ fitted detailed kinetic models using relatively inefficient MCMC algorithms that do not scale well to high dimensional parameter spaces, limiting the scope of modeling. Conversely, St. John et al.^17^ utilizes an efficient sampling algorithm but uses approximate kinetics, namely, lin-log kinetics,^18^ limiting the scope of interpreting parameters and inferring cellular behavior in experimental conditions outside the reference data set.

There have also been efforts to implement Bayesian inference for kinetic models without the use of MCMC. Examples of alternative inference methods include variational inference,^17^ rejection sampling, and approximate Bayesian computation^7^ and Laplace approximation, in which the Fisher information matrix is used to calculate a normal approximation around the maximum a posteriori parameter configuration^8,15,16,19^ Non-MCMC-based Bayesian kinetic models suffer from a lack of reliable diagnostic tools for verifying that their results approximate the target posterior distribution. This is a problem because realistic kinetic models tend to induce highly correlated, non-Gaussian, joint probability distributions.^9,16^

Our application Maud provides a Bayesian kinetic model that combines biologically realistic mechanistic accuracy—including accurate rate laws, post-translational modification, and thermodynamics—with fast, robust MCMC sampling using adaptive Hamiltonian Monte Carlo. Further, Maud is a general-purpose application that can be used to fit a wide range of Bayesian kinetic models.

## 2 Results and Discussion

To demonstrate our application’s capabilities, we used Maud to analyze an artificial data set based on the human methionine cycle. We generated this data set using Maud, by simulating measurements for a set of training and validation conditions based on plausible parameter values. Next, we performed posterior inference for the training measurements and a prediction for both training and validation measurements.

We investigated Maud’s sensitivity to missing measurements by comparing the results of fitting a full data set with an intentionally incomplete data set. To demonstrate why a full Bayesian approach is preferable to an approach based on a Laplace approximation of the posterior distribution, we compared the results of analyzing a representative metabolic network using both methods.

Finally, we dug into our results to find out what our full data set methionine model learned about the contributions of different regulatory factors to the flux through GNMT, an important reaction. This analysis illustrates how Maud can be used to generate actionable insights into metabolism without the need for further statistical analysis.

**Figure 1:**
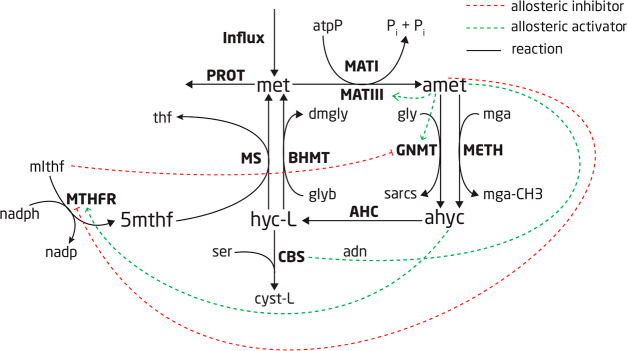
Methionine cycle as modeled, with the solid black lines representing the reactions, the green lines representing allosteric interaction, and the red lines representing allosteric inhibition. The bold fonts are the reaction names and the regular font represents the metabolites.

The methionine cycle, illustrated in Figure 1, is a fundamental pathway in human metabolism, whose intermediate metabolites participate in a variety of mechanisms that must compete for the same resources. Due to this competition, as well as the fact that all the functions occur simultaneously, the methionine cycle is highly regulated, with 6 known allosteric effectors.^20−22^ This complex regulation means that quantitative modeling of the methionine cycle requires a detailed kinetic model: this is why we chose it as a case study for Maud.

### 2.1 Data Set and Model Specification

The simulated data set and underlying kinetic model that we used for our analysis can be found at https://github.com/biosustain/Methionine_model/tree/main/data/methionine and is described in Supporting Information Section 3.

We constructed a kinetic model of the methionine cycle in Maud’s format using the description in Korendyaseva et al.^22^ The ordinary differential equation system describing this model is
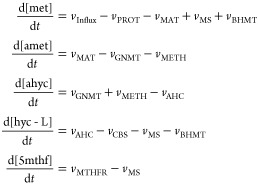
4

After specifying the qualitative aspects of the kinetic model, we selected parameter values to use as ground truth by Monte Carlo sampling using a previous model of the methionine cycle as a starting point (see Saa and Nielsen^7^ for this model).

We used these parameters to simulate steady states in a range of plausible experimental conditions, again using Saa and Nielsen^7^ as a starting point. These steady states were then used to generate simulated measurements by using the measurement model.

For enzyme and metabolite concentration measurements, we specified a standard deviation of 0.1 on a natural logarithmic scale, corresponding to approximately 10% measurement error. For each reaction measurement, the measurement standard deviation was approximately 10% of the simulated value.

These measurement error specifications are somewhat optimistic considering the many sources of variation and uncertainty affecting quantitative proteomics, metabolomics, and fluxomics analyses, but are a reasonable first approximation to a realistic set of measurements.

For our main model run, we assumed that all metabolite and enzyme concentrations were measured and that there was a reaction measurement for each of the network’s free fluxes.

The simulated experiments and measurements were split into a training and a validation data set in a way that achieved a large difference in flux between the two categories. This was done to evaluate whether the fitted model is able to extrapolate to conditions well outside the training data set rather than merely interpolating between the training data without necessarily learning the system.

We constructed inputs in Maud’s format for each of the analyzed data sets, based on the scenario that the true kinetic model was known except for parameter values, which needed to be inferred from the training data and priors. These inputs can be found at https://github.com/biosustain/Methionine_model/tree/main/data.

### 2.2 Posterior Inference

The prior distributions and corresponding true parameter values used in our case study are shown in Supporting Information Section 3.2. They were chosen to reflect a plausible pre-experimental information state. In 7 cases, the marginal prior distribution for a parameter disagrees with the true parameter values used to generate the data; a similar situation is likely to occur in practice due to in vivo vs in vitro measurement differences.

Running standard diagnostic checks indicated that the samples we generated were from the target posterior distribution. The improved *R̂* statistic^14^ for every variable of interest was within 2% of 1, indicating appropriate mixing within and between Markov chains. Additionally, the number of effective samples was high, indicating that we generated enough posterior samples to support inferences about the bulk of the distributions of the sampled parameters. Furthermore, we observed no postwarmup divergent transitions, indicating that the sampler was able to transform the log-posterior distribution, avoiding any regions with excessive curvature that might inhibit exploration via HMC.

**Figure 2:**
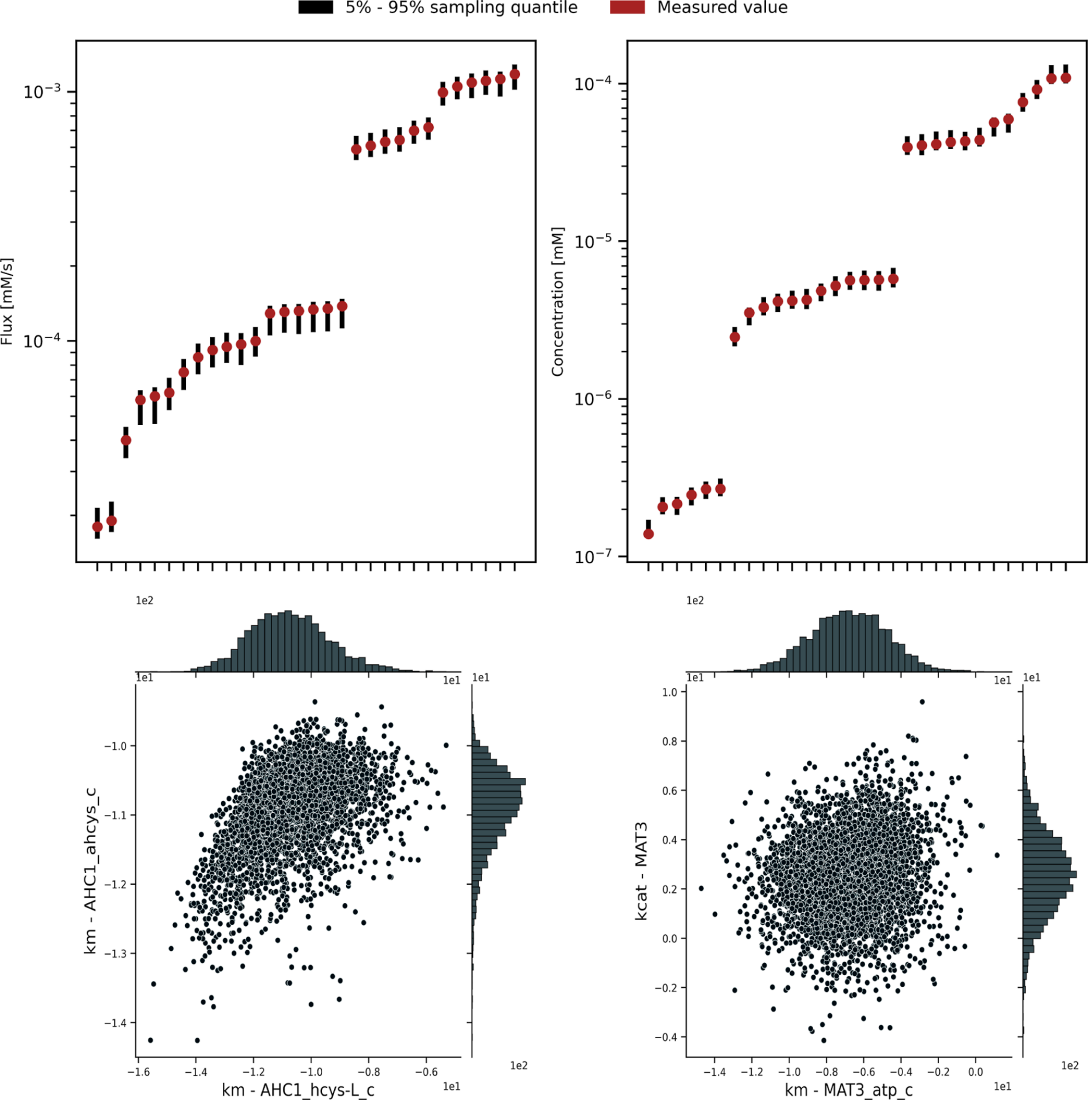
Marginal posterior distributions from our main model run. (Top left) Comparison of posterior predictive intervals with simulated flux measurement values. All the flux measurements are within the predictive intervals, indicating a good fit. (Top right) Comparison of posterior predictive intervals with simulated concentration measurement values. These also show a good fit. (Bottom left) Pairwise marginal posterior distribution for two correlated parameters, namely, Km^AHC1,hcys-L^ and . (Bottom right) Pairwise marginal posterior distribution for two uncorrelated parameters, namely, Km^MAT3,atp^ and K_cat_^MAT3^.

Posterior predictive checking indicated that our model achieved a good fit to the simulated reaction and metabolite concentration measurements, as shown by the graphs in the top row of Figure 2.

Analysis of the posterior distributions for the kinetic parameters indicated that these are highly correlated. The marginal posterior distributions for most kinetic parameters did not shrink significantly compared to the corresponding marginal prior distributions, even though these parameters’ joint posterior distribution contained enough information to make accurate out of sample predictions. In some cases, there were two-dimensional correlations such as the one shown in the bottom left of Figure 2; in this case, the marginal distribution of the two parameters is roughly banana-shaped. More commonly, however, two-dimensional pair plots were insufficient to reveal the underlying correlation structure, as seen in the bottom-right plot in Figure 2, which shows two marginally independent parameters.

These results show that Maud can achieve an adequate fit to a realistic pathway-sized data set. This was achieved without fixing the marginal values of the kinetic parameters: the information required to make good predictions was contained in the correlation structure of the joint posterior distribution. This finding is consistent with previous analyses of biological systems that found they are “sloppy”, that is, sensitive to parameter combinations rather than marginal parameter values, with important combinations, scales, and regions of sensitivity being difficult to ascertain in advance.^9,23^

The question naturally arises whether the crucial high-dimensional parameter correlations are linear or nonlinear. This is relevant to the question of model performance as linear correlations are easier to correct for. A linearly correlated posterior space would also be easier to summarize. We address this question in the next section.

#### 2.2.1 Comparison with Laplace Approximation

This current case study illustrates the type of kinetic model and data set that Maud can fit. The model we analyzed has 10 reactions, 5 state variables, and 212 parameters. Generalizing from our ability to fit this model in a reasonable time using Maud, we expect that Maud can be used to fit realistic Bayesian models of approximately the same size but not, for example, genome-scale kinetic models. To fit larger models, faster steady-state solving methods or alternative inference algorithms will be required. The Laplace approximation, in which the Fisher information matrix is used to calculate a normal approximation around the maximum a posteriori parameter configuration, is a popular strategy for generating approximate posterior samples while avoiding a full MCMC approach.

Using the recently implemented Laplace approximation in Stan, we were unable to generate approximate posterior samples for our main methionine cycle case study using the Laplace approximation, as the algorithm could not recover from solver failures caused by unrealistic parameter configurations. This is not an inherent issue of the Laplace transformation but highlights some of the challenges around solving^3^ for real problems.

In order to assess the potential use of the Laplace approximation, we made a comparison for a simpler model. This input can be found at https://github.com/biosustain/Methionine_model/tree/main/data/example_ode. MCMC sampling for this simpler model yielded 800 samples in 625 min, while Laplace sampling yielded the same number of samples in only 1 min. The diagnostics indicated that our algorithm was able to find the maximum a posteriori parameter configuration, approximate the Hessian, and use these quantities to generate approximate posterior samples. The results can be found at https://github.com/biosustain/Methionine_model/tree/main/results.

**Figure 3:**
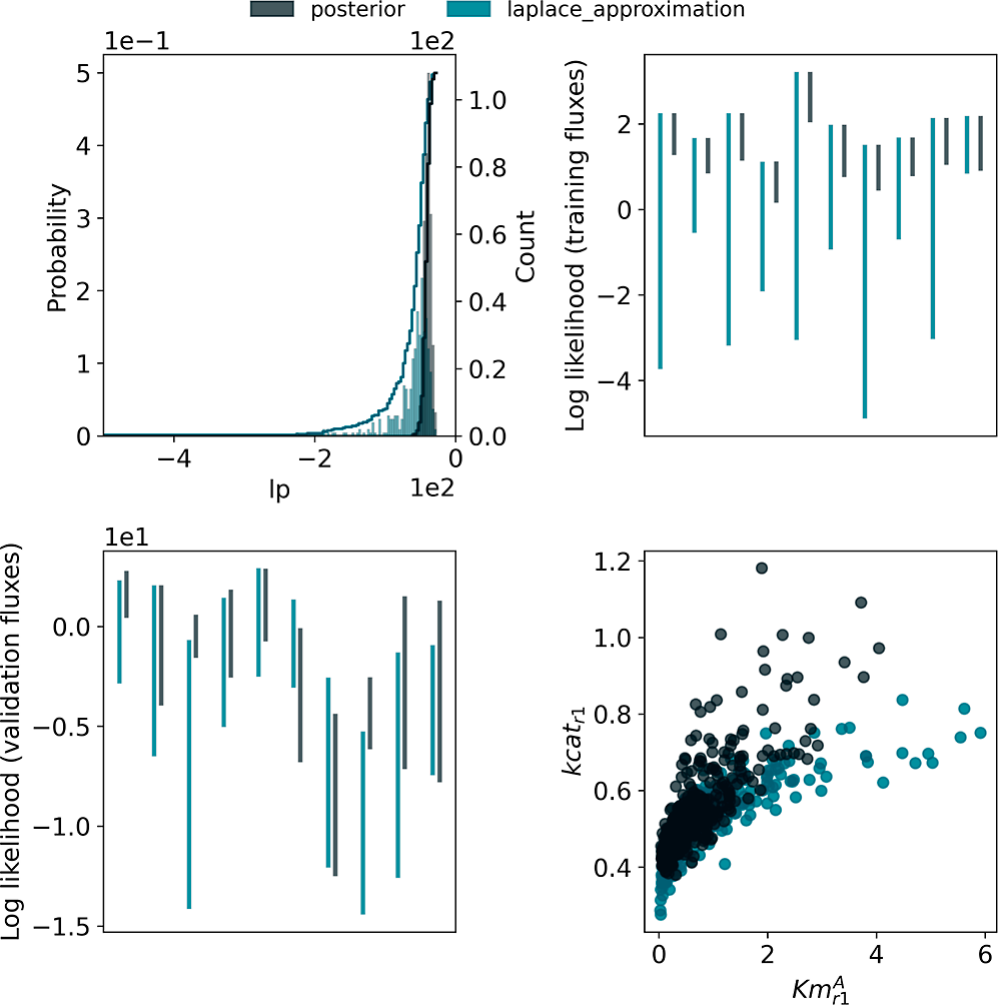
Graphical comparison of approximate posterior samples generated using Laplace sampling (blue-green) with posterior samples generated using MCMC (dark gray). (Top left) The two sets of samples clearly have different marginal distributions for the overall log probability variable, indicating that the Laplace samples do not accurately approximate the target distribution. (Top right) The distribution of marginal posterior predictive log likelihood values for training data flux measurements shows that the Laplace method tended to yield much worse predictions compared with the true model (lower log likelihood values indicate that the modeled and measured values are further away). (Bottom left) The Laplace method also tended to produce worse flux predictions for held-back test measurements. (Bottom right) The marginal joint distribution of two parameters: K_m_^*A*,*r*1^ and K_cat_^*r*1^. The Laplace method is not able to track the correct joint distribution for this pair of parameters. This is unsurprising given that the target distribution has position-dependent scales, which are difficult for a linear approximation to capture.

Comparing the samples generated using each method shows that the Laplace approximation does not provide a good approximation to the true posterior distribution in this case (Figure 3). As can be seen from the top left plot, the total log probability density is clearly different and this was confirmed by a test of the equality of two empirical univariate distributions (Kolmogorov–Smirnov, *p* = 1.7 × 10^–65^).

The difference between the Laplace approximation output and the true posterior distribution manifests not only in the parameter space but also in the measurement space for both training data (upper right) and validation data (lower left). The lower log likelihood values indicate that the modeled and measured values are further away and that the Laplace approximation yielded significantly worse predictions than the true posterior, even for the training data.

To further explore why this is the case, we compared samples from the true posterior and the Laplace approximation for the pairwise marginal distributions of two Michaelis–Menten constants *K*_m_^*A*,*r*1^ and *K*_cat_^*r*1^ (Figure 3, bottom right). This comparison demonstrates that the Laplace method is not able to capture the correct relationships between parameters’ distributions.

This result shows that MCMC, while slower than the Laplace approximation, is required for posterior inference in this case. We expect that the Laplace method will produce worse approximations the more complex the target model. Since the approximation is already unacceptable for our simple test model, we recommend that Maud users use MCMC sampling in preference to Laplace approximation if possible when fitting realistic Bayesian kinetic models.

Our results here also provide circumstantial evidence that the parameter correlations in Bayesian kinetic model posteriors tend to be nonlinear, as a posterior with only linear correlations would likely be more germane to Laplace approximation. A conclusion that we drew from this analysis was that the results of fitting our model cannot be summarized simply, for example, by fitting a multivariate normal distribution to the posterior draws. We therefore recommend that Maud users store the full set of MCMC drawings rather than use such an approximation. This does not preclude the possibility that there is an alternative, more compact, way to summarize the results of Bayesian kinetic model inference; we leave research into this topic to future work.

### 2.3 Effect of Missing Metabolite Concentration Measurements

To gain insight into our model’s robustness to missing measurements, we also performed a model run with the same 6 experimental data sets, but with measurements of the metabolite *S*-adenosyl-l-homocysteine, or “ahcys” removed. Since ahcys regulates three enzymes in the methionine cycle, including one enzyme, which is also thermodynamically regulated, we expected the removal of these measurements to yield interesting results.

Comparing model runs with and without the ahcys measurements showed that Maud can produce sensible results, even from incomplete metabolomics data.

**Figure 4:**
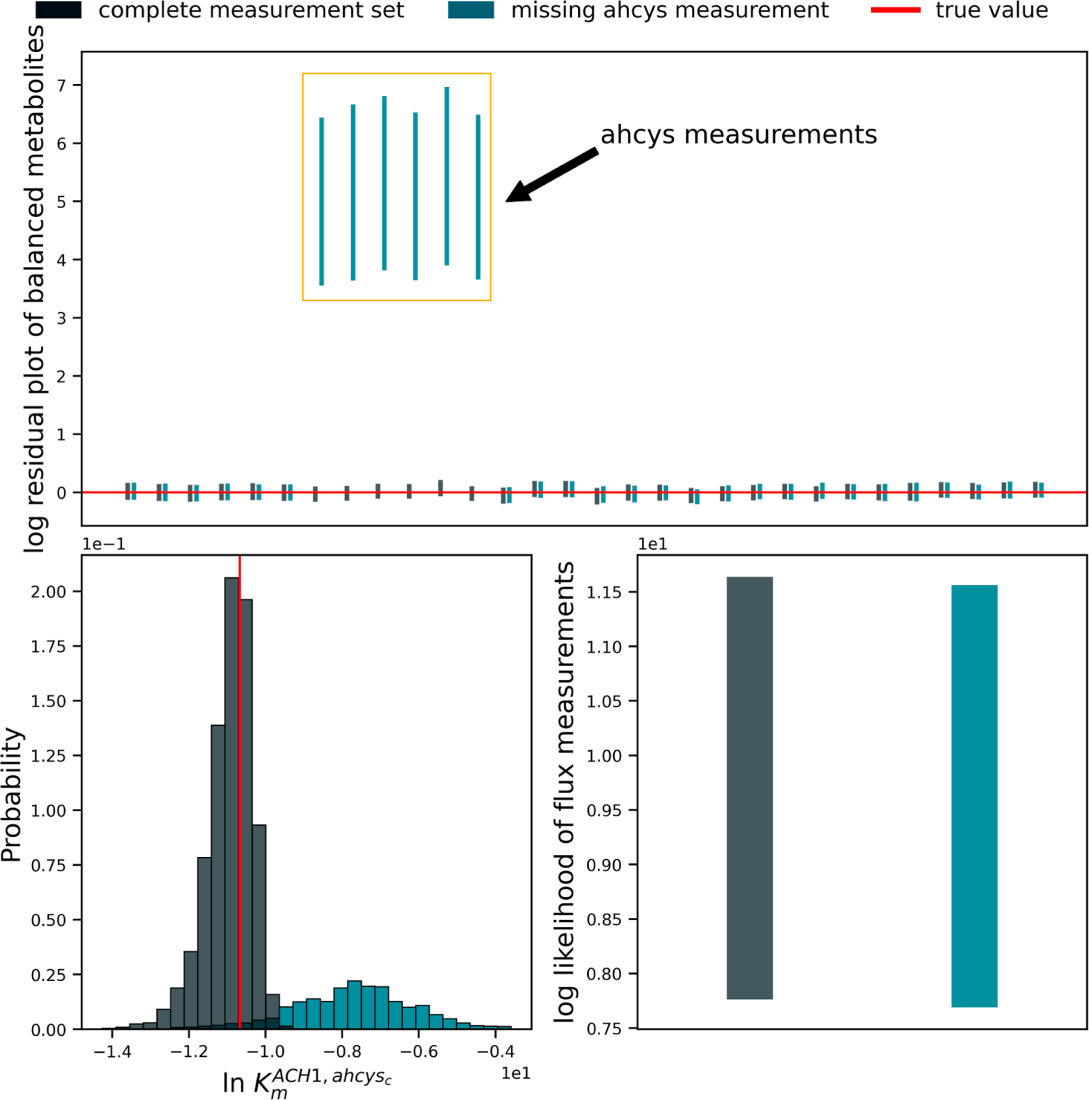
Results of removing concentration measurements for the metabolite ahcys_c_ from our case study data set. (Top) Comparison of metabolite concentration residuals between the full measurement data set (blue-green) and the missing-data data set (gray), displayed on natural logarithmic scale. The missing-data model was unable to estimate the withheld ahcys_c_ concentrations. (Bottom Left) The marginal posterior distribution for the Michaelis constant  in each model, alongside the true parameter value used to generate both data sets. The true value is recovered by the complete-data model but not by the missing-data model. (Bottom Right) The distribution of total log-likelihood for out-of-sample flux measurements in both models. There is a significant overlap between the two distributions, suggesting that removing the *ahcys*_*c*_ measurement did not cause catastrophic prediction failure. However, overall, the complete-data model tended to make better predictions.

As might be expected, the model with missing measurements did not correctly infer the missing ahcys concentrations (Figure 4). Nonetheless, the remaining measured metabolites were still well predicted, suggesting that information about the network is still preserved despite the missing measurements. Comparison of flux measurements in both models also indicated that removing the ahcys measurement did not result in a catastrophic model failure.

The missing measurements did affect Maud’s ability to infer parameter values correctly (Figure 4, lower left). The model with full metabolomics learned the true value for the displayed dissociation constant despite this value being far from the mean of the corresponding marginal prior distribution. In contrast, the model with missing measurements stayed in the neighborhood of the prior.

This result is reassuring because not having access to all measurements is a common situation in multiomics studies. For instance, measuring all metabolites in a pathway can be infeasible because of limitations of mass spectrometers, availability of standards, column effects, and compartmentalization. However, provided that sufficient information is available from other sources, our approach can produce sensible results from incomplete metabolomics data.

### 2.4 Application to Regulatory Understanding

**Figure 5:**
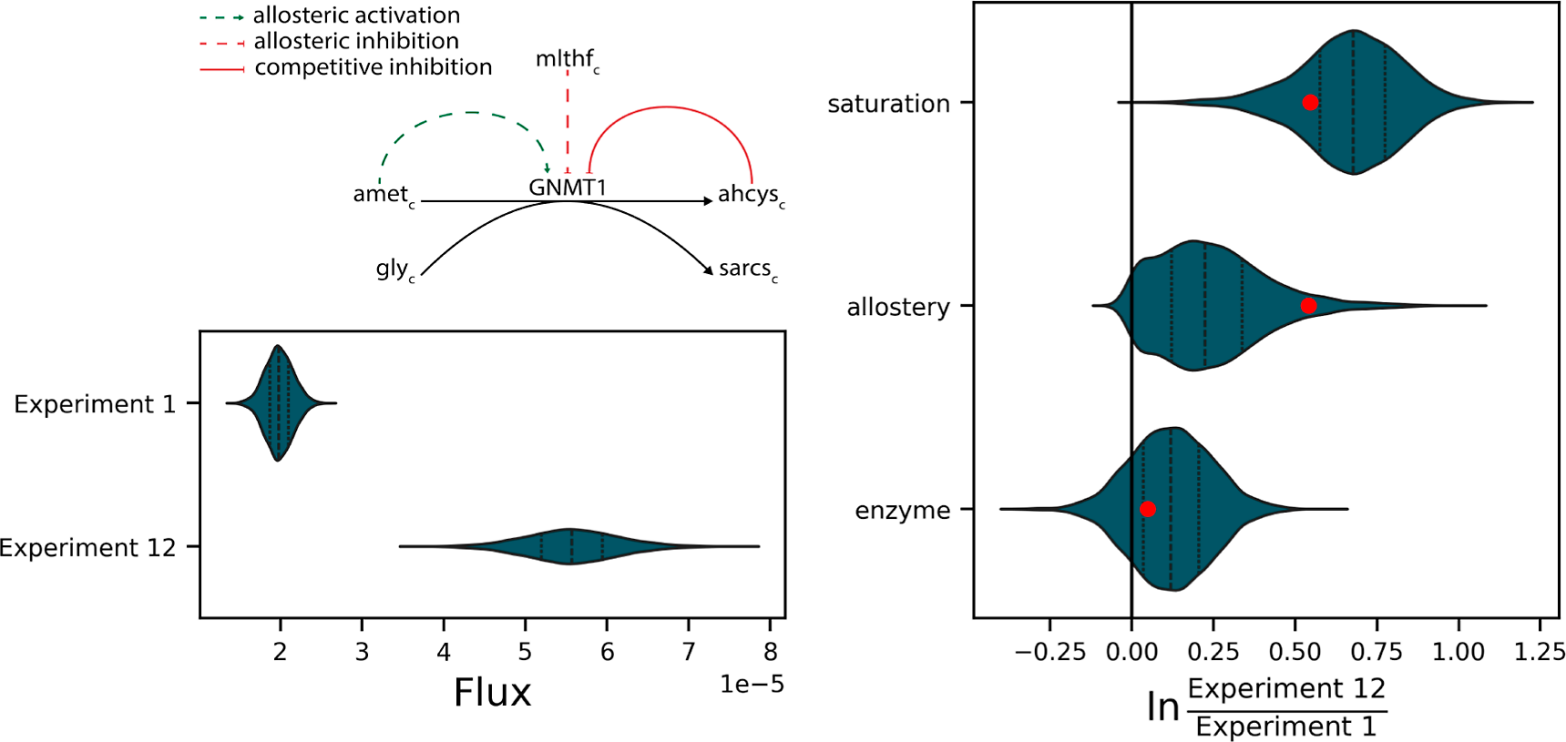
Illustration of how analyzing a system with Maud can yield actionable insights into the underlying metabolic network. (Top Left) Schematic of the regulatory interactions associated with the enzyme *GNMT*1. Dashed green lines represent allosteric activation, dashed red lines indicate allosteric inhibition, and solid red lines represent competitive inhibition. (Bottom Left) Comparison of marginal posterior distributions for *GNMT* flux in data sets 1 and 2. (Right) Log-scale ratios of the regulatory elements defined in ref (5). Note that the reversibility and *k*_cat_ components are excluded: this is because this reaction was modeled as irreversible, and *k*_cat_ was modeled as constant across data sets. These plots identify why flux in data set 12 is higher than in data set 1: the flux increase is due to allostery and saturation with no control from enzyme concentration changes.

To demonstrate how Maud’s output can be used to yield useful metabolic insights, we used the results of our case study to explain why the flux of the enzyme GNMT is higher in data set 1 than in data set 12 (Figure 5). GNMT is an irreversible enzyme that is homotropically activated by its substrate, competitively inhibited by its product, and heterotropically inhibited by 5,10-methylenetetrahydrofolate (mlthf). The complex regulation makes it the ideal test case to elucidate regulatory changes.

Regulation can be separated into enzyme abundance, allostery, and saturation, and we can plot the marginal posterior distribution of the ratio of the corresponding regulatory component in data set 1 compared with data set 12 (Figure 5, right panel). A positive value indicates that the component was increased in data set 1 relative to data set 12, with 0 indicating no difference. The probability, according to our model, of the component acting in each direction is given by the relative area under the curve on each side of the zero point.

Our model correctly inferred that saturation and allosteric effects were the main drivers of regulation between the two data sets in this case, with the curves for each component aligning with the ground truth shown in red.

Importantly, this form of analysis takes into account all modeled sources of uncertainty, including uncertainty about the measured values of the flux in each data set. Our result shows that Maud could be used in this realistic case not only to explain an observed difference in fluxes but also to provide a reasonable judgment as to the explanation’s robustness.

## 3 Methods

Maud is a command line application implementing Bayesian inference for a wide range of realistic kinetic models. Maud is written in Python,^24^ designed for use on Windows, macOS, and Linux, is pip installable from the Python Package Index as maud-metabolic-models, documented at https://maud-metabolic-models.readthedocs.io and actively developed and maintained at https://github.com/biosustain/Maud/.

To use Maud, a user must first collate appropriate input information, represent it in files with Maud’s required formats (see the Supporting Information section on input format). Maud’s command line interface provides commands for inference via a range of algorithms including adaptive Hamiltonian Monte Carlo as well as commands for simulation and making out-of-sample predictions. Results are stored in files using a structured, interoperable format.

As well as parameter values, Maud also performs inference for derived quantities including the components of the regulatory decomposition described below in,^5^ log likelihoods, simulated measurement values, and metabolic control analysis coefficients.^25^

In the rest of this section, we describe Maud’s kinetic model at a high level and discuss Maud’s statistical model, implementation, and how it solves the crucial steady state problem. Further details about Maud’s kinetic model can be found in the Supporting Information.

### 3.1 Kinetic Model

Maud’s kinetic model decomposes into factors
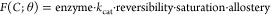
5

Each of the terms on the right-hand side of^5^ is a function of *C* and θ. This idea is taken from Noor et al.^26^ The terms usefully gather physically meaningful and conceptually distinct factors contributing to the reaction fluxes. The enzyme captures the effect of enzyme concentration, *k*_cat_ that of enzyme efficiency, reversibility quantifies thermodynamic effects, saturation the effect of enzyme availability, and allostery the effect of post-translational modifications.

We used the model of enzyme saturation from Liebermeister et al.^27^ and the generalized Monod–Wyman–Changeux model of Allosteric regulation introduced in^28−31^ and used more recently in Matos et al.^32^ To capture the effect on enzyme activity of coupled phosphorylation and dephosphorylation processes, we developed a new mathematical model inspired by the generalized MWC model of allosteric regulation. Full details of all mathematical aspects of Maud’s kinetic model can be found in Supporting Information Section 2.

### 3.2 Statistical Model

Maud used MCMC to perform posterior inference on a Bayesian statistical model. This section introduces these topics and then describes exactly what kind of Bayesian statistical model Maud uses.

#### 3.2.1 Bayesian Inference

Bayesian statistical inference analyzes data by constructing a mathematical model with the following three components:

- A measurement model or “likelihood” that probabilistically describes how likely any possible measurement set is given the true values of the measured quantities, i.e. a probability density function  where  is the set of all possible measurement sets and  is the set of all possible true measurable values.
- A deterministic process model that describes the measured quantities in terms of unknown, possibly unobserved parameters θ, i.e. a function .
- A prior model that probabilistically describes how likely any possible set of parameter values is, without considering any information included in the measurement model, i.e. a probability density function π: θ → [0, 1].

Together these components determine a joint probability function that encapsulates the Bayesian statistical model, i.e. a function  such that for any θ and *y*, *p*(θ, *y*) = π(θ)*l*(*y*|θ). Substantive questions about the implications of the assumptions implicit in the model components can be formulated in terms of this joint density function.

In particular, questions about what the model implies, given a particular measurement set, *y* can be formulated in terms of the conditional probability density function *p*_|*y*_:θ → [0,1]. Bayes’s theorem guarantees that this conditional density is proportional to the product of the prior and likelihood, i.e., *p*_|*y*_(θ) ∝ π(θ)*l*(*y*|θ) see (ref (11), Ch 1) for an extended discussion of the theory of Bayesian statistical inference.

The principal computational challenge for Bayesian statistical inference is to approximate the values of integrals of the joint density function conditional on a known measurement assignment *y*, also known as the posterior distribution. For most nontrivial statistical models, the posterior distribution cannot be integrated analytically, so numerical approximation is required. MCMC is a popular method for addressing this problem, which uses a Markov chain with known properties to generate samples from a target probability distribution that can be used to perform Monte Carlo integration. Hamiltonian Monte Carlo makes it possible, given an appropriate choice of hyperparameters, to efficiently generate samples from even a high-dimensional continuous posterior distribution by calculating the gradients of the log-scale joint density function. Adaptive Hamiltonian Monte Carlo, as used by Stan, uses a range of well-tested methods to tune these hyperparameters, allowing efficient MCMC for high-dimensional posterior distributions with minimal user input.

Alternatives to MCMC for numerically approximating integrals over posterior distributions include grid sampling (11, Ch. 10), rejection sampling (11, Ch. 10), importance sampling,^33^ and distributional approaches including variational inference and Laplace approximation (11, Ch. 13).

The Laplace approximation is of particular interest because, as mentioned above, this has been used for approximate Bayesian inference in a similar context to Maud. Laplace approximation of a posterior distribution works by first finding the mode of the posterior distribution, i.e., the “maximum a posteriori” parameter configuration with the highest posterior density. Next, the second derivative of the posterior distribution at this point is found and used to generate a normal distribution that either approximates the target distribution or can in turn be used to generate such an approximation.

Maud employs adaptive Hamiltonian Monte Carlo to perform posterior inference for a Bayesian statistical model, where the process model is the kinetic model described above. The next two subsections describe Maud’s prior and measurement models.

#### 3.2.2 Prior Model

Maud’s prior model includes unknown parameters corresponding to quantities in the kinetic model that are assumed to be unknown, other than steady-state metabolite concentrations and fluxes, which are derived from the values of other parameters by solving the steady-state problem. See Table 1 in this paper’s Supporting Information for a description of all these parameters and their dimensions.

Except for metabolites’ standard condition Gibbs energy changes of formation, Maud uses independent normal prior distributions for parameters that can in principle be both negative and positive. For parameters that are constrained to be positive, Maud et al. use independent log–normal distributions. Formation energy parameters have a multivariate normal prior distribution. Location, scale, and covariance parameters for all these prior distributions can be selected freely by the user.

#### 3.2.3 Measurement Model

Maud’s measurement model considers three types of measurement: metabolite concentration measurements, enzyme concentration measurements, and flux measurements, represented by vectors *y*^conc^, *y*^enz^, and *y*^flux^, respectively.

All measurements are specific to an experimental condition; that is, a case where the true state of the network, including knockouts, boundary conditions, and state variables as well as kinetic and thermodynamic parameters, can safely be assumed to be the same. Maud’s statistical model allows for arbitrarily many experimental conditions and for any measurable quantity to be measured any number of times in any condition.

Metabolite and enzyme measurements are intended to represent the results of quantitative metabolomics and proteomics experiments. The likelihood functions for such measurements are
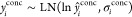
6
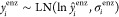
7

Both equations are log–normal generalized linear models with a standard link function (the natural logarithm ln) and known standard deviation σ_conc_. The use of this measurement model is motivated by the consideration that concentrations are constrained to be non-negative, so the measurement model should avoid assigning positive probability mass to negative metabolite concentration values. In addition, we expect the precision of most metabolomics and proteomics experiments to be roughly proportional to the true value of the measured quantity, which supports a measurement model with constant coefficient of variation. The measurement standard deviations σ_conc_ and σ_enz_ are assumed to be known exactly for the sake of simplicity; plausible values can be elicited by considering the likely coefficient of variation of the measuring apparatus. Our measurement model improves on analyses of metabolomics and proteomics data that assume a regression model with normally distributed errors, whether explicitly using a standard linear model or implicitly using ordinary least-squares fitting.

Flux measurements, representing the results of quantitative fluxomics analyses, are modeled using a likelihood function from a standard linear regression model.^8^ Flux measurements can be obtained from isotope labeling experiments using metabolic flux analysis, for example, as described in (Young 2014). When entering flux measurements, it is important only to specify measurements for a network’s free fluxes, as the values of some steady state fluxes in a metabolic network are constrained by others, with the result that dependent fluxes cannot typically be measured separately. If measurements of multiple dependent fluxes are entered, information will inappropriately be double counted.
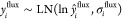
8

The use of independent measurement models for metabolite, enzyme, and flux measurements carries an implicit assumption that there are no systematic correlations in the measurement errors. This choice was motivated by simplicity; it would be better to use a model with potentially correlated measurements. Similarly, it would be preferable to include measurement errors as model parameters, thereby avoiding possible bias due to incorrect assessments of the measurement accuracy. However, we chose to use a simpler measurement model to avoid the complexity and potential fitting issues that these changes would entail.

Finally, the reader may wonder why Maud uses a linear regression model for reaction flux measurements even though this creates the potential for erroneous double counting and requires nontrivial upstream modeling, as intracellular fluxes typically cannot be measured directly. Instead, fluxes are typically inferred from isotope labeling experiments using metabolic flux analysis: see Dai and Locasale^34^ for more about this method. Ideally, Maud’s measurement model for fluxes would extend from fluxes to the results of potential labeling experiments, thereby removing the need for upstream analysis and avoiding any double counting. This option has not yet been pursued, again for the sake of simplicity.

### 3.3 Implementation

Maud uses the Python library click^35^ to implement a command line interface. The command line interface loads input files as Python dictionaries, which are parsed using the Python library toml^36^ and then validated and converted into structured MaudInput objects using Pydantic.^37^ Maud’s statistical model is implemented in the probabilistic programming language Stan^38^ and accessed using the interface cmdstanpy.^39^ For posterior sampling, Maud uses MaudInput to create an input file for Stan and then triggers posterior sampling using adaptive Hamiltonian Monte Carlo. See Betancourt^40^ for more about this algorithm.

When sampling is complete, Maud converts to the output into the standard format InferenceData using the Python library arviz^41^ and saves it as a json file, along with some information for debugging. This format allows for easy checking of MCMC diagnostics including divergent transitions, effective sample size, and the improved *R̂* statistic proposed in Vehtari et al.^14^

### 3.4 Solving the Steady-State Problem

Maud’s parameters are connected with measurable quantities via the steady-state equation.^3^ Posterior sampling using adaptive Hamiltonian Monte Carlo requires repeatedly solving^3^ and finding the gradients of its solution with respect to all model parameters. This must be done numerically, as analytic solutions are not available for realistic kinetic models.

The speed at which this problem can be solved is tightly coupled with the size and complexity of metabolic network that can practically be modeled. See Timonen et al.^42^ for more about considerations involved in this kind of modeling.

To solve^3^ and find its gradients, Maud uses a hybrid method involving two numerical solvers from the SUNDIALS suite:^43^ CVODES and IDAS via their interface from Stan. The hybrid method follows that proposed by Margossian^44^ and involves numerically evolving the ODE system for a short period of time and then using the difference between the evolved and starting concentrations as the target for a numerical algebra solver.

## Data Availability

The analyses described in this paper, as well as instructions for reproducing our results, figures and paper, can be found at https://github.com/biosustain/Methionine_model.

## Supporting Information Available

Description of Maud’s input format and Maud’s kinetic model including rate equations as well as parameters and their dimensions; details of the procedure used to generate our case study results, including generating the artificial case study data sets, specifying priors and carrying out computation; and specification of the prior distribution used for the case studies (PDF)

## References

[ref1] Christodoulou D.; Link H.; Fuhrer T.; Kochanowski K.; Gerosa L.; Sauer U. Reserve Flux Capacity in the Pentose Phosphate Pathway Enables Escherichia coli’s Rapid Response to Oxidative Stress. Cell Syst. 2018, 6, 569–578.e7. 10.1016/j.cels.2018.04.009. 29753645

[ref2] DeBerardinis R. J.; Chandel N. S. Fundamentals of Cancer Metabolism. Sci. Adv. 2016, 2, e1600200 10.1126/sciadv.1600200. 27386546 PMC4928883

[ref3] Liberti M. V.; Dai Z.; Wardell S. E.; Baccile J. A.; Liu X.; Gao X.; Baldi R.; Mehrmohamadi M.; Johnson M. O.; Madhukar N. S.; et al. A Predictive Cell Syst.for Selective Targeting of the Warburg Effect through GAPDH Inhibition with a Natural Product. Cell Metab. 2017, 26, 648–659.e8. 10.1016/j.cmet.2017.08.017. 28918937 PMC5629112

[ref4] Siskos A. P.; Jain P.; Römisch-Margl W.; Bennett M.; Achaintre D.; Asad Y.; Marney L.; Richardson L.; Koulman A.; Griffin J. L.; Raynaud F.; Scalbert A.; Adamski J.; Prehn C.; Keun H. C. Interlaboratory Reproducibility of a Targeted Metabolomics Platform for Analysis of Human Serum and Plasma. Anal. Chem. 2017, 89, 656–665. 10.1021/acs.analchem.6b02930. 27959516 PMC6317696

[ref5] Tabb D. L.; Vega-Montoto L.; Rudnick P. A.; Variyath A. M.; Ham A. J. L.; Bunk D. M.; Kilpatrick L. E.; Billheimer D. D.; Blackman R. K.; Cardasis H. L.; et al. Repeatability and Reproducibility in Proteomic Identifications by Liquid Chromatography—Tandem Mass Spectrometry. J. Proteome Res. 2010, 9, 761–776. 10.1021/pr9006365. 19921851 PMC2818771

[ref6] Lu W.; Su X.; Klein M. S.; Lewis I. A.; Fiehn O.; Rabinowitz J. D. Metabolite Measurement: Pitfalls to Avoid and Practices to Follow. Annu. Rev. Biochem. 2017, 86, 277–304. 10.1146/annurev-biochem-061516-044952. 28654323 PMC5734093

[ref7] Saa P. A.; Nielsen L. K. Construction of feasible and accurate kinetic models of metabolism: A Bayesian approach. Sci. Rep. 2016, 6, 29635 10.1038/srep29635. 27417285 PMC4945864

[ref8] Gopalakrishnan S.; Dash S.; Maranas C. K-FIT: An accelerated kinetic parameterization algorithm using steady-state fluxomic data. Metab. Eng. 2020, 61, 197–205. 10.1016/j.ymben.2020.03.001. 32173504

[ref9] Gutenkunst R. N.; Waterfall J. J.; Casey F. P.; Brown K. S.; Myers C. R.; Sethna J. P. Universally sloppy parameter sensitivities in systems biology models. PLoS Comput. Biol. 2007, 3, e189–e1878. 10.1371/journal.pcbi.0030189. 17922568 PMC2000971

[ref10] Raue A.; Becker V.; Klingmüller U.; Timmer J. Identifiability and observability analysis for experimental design in nonlinear dynamical models. Chaos 2010, 20, 045105 10.1063/1.3528102. 21198117

[ref11] Gelman A.; Carlin J. B.; Stern H. S.; Dunson D. B.; Vehtari A.; Rubin D. B. Bayesian Data Analysis, 3rd ed.; CRC Press, 2020.

[ref12] Gelman A.; Vehtari A.; Simpson D.; Margossian C. C.; Carpenter B.; Yao Y.; Kennedy L.; Gabry J.; Bürkner P.-C.; Modrák M. Bayesian Workflow. arXiv 2020, 2011.01808 [stat.ME] 10.48550/arXiv.2011.01808.

[ref13] White A.; Tolman M.; Thames H. D.; Withers H. R.; Mason K. A.; Transtrum M. K. The limitations of model-based experimental design and parameter estimation in sloppy systems. PLoS Comput. Biol. 2016, 12, e1005227 10.1371/journal.pcbi.1005227. 27923060 PMC5140062

[ref14] Vehtari A.; Gelman A.; Simpson D.; Carpenter B.; Bürkner P. C. Rank-Normalization, Folding, and Localization: An Improved R̂for Assessing Convergence of MCMC (with Discussion). Bayesian Anal. 2021, 16, 667–718. 10.1214/20-ba1221.

[ref15] Raue A.; Kreutz C.; Theis F. J.; Timmer J. Joining forces of Bayesian and frequentist methodology: a study for inference in the presence of non-identifiability. Philos. Trans. R. Soc., A 2013, 371, 20110544 10.1098/rsta.2011.0544. 23277602

[ref16] Stapor P.; Weindl D.; Ballnus B.; Hug S.; Loos C.; Fiedler A.; Krause S.; Hroß S.; Fröhlich F.; Hasenauer J.; Wren J. PESTO: parameter estimation toolbox. Bioinformatics 2018, 34, 705–707. 10.1093/bioinformatics/btx676. 29069312 PMC5860618

[ref17] St. John P. C.; Strutz J.; Broadbelt L. J.; Tyo K. E. J.; Bomble Y. J. Bayesian Inference of Metabolic Kinetics from Genome-Scale Multiomics Data. PLoS Comput. Biol. 2019, 15, e1007424 10.1371/journal.pcbi.1007424. 31682600 PMC6855570

[ref18] Visser D.; Heijnen J. J. Dynamic simulation and metabolic re-design of a branched pathway using linlog kinetics. Metab. Eng. 2003, 5, 164–176. 10.1016/S1096-7176(03)00025-9. 12948750

[ref19] Liebermeister W.; Noor E. Model Balancing: A Search for In-Vivo Kinetic Constants and Consistent Metabolic States. Metabolites 2021, 11, 749 10.3390/metabo11110749. 34822407 PMC8621975

[ref20] Martinov M. V.; Vitvitsky V. M.; Mosharov E. V.; Banerjee R.; Ataullakhanov F. I. A Substrate Switch: A New Mode of Regulation in the Methionine Metabolic Pathway. J. Theor. Biol. 2000, 204, 521–532. 10.1006/jtbi.2000.2035. 10833353

[ref21] Nijhout H.; Reed M. C.; Anderson D. F.; Mattingly J. C.; James S. J.; Ulrich C. M. Long-Range Allosteric Interactions between the Folate and Methionine Cycles Stabilize DNA Methylation Reaction Rate. Epigenetics 2006, 1, 81–87. 10.4161/epi.1.2.2677. 17998813

[ref22] Korendyaseva T. K.; Kuvatov D. N.; Volkov V. A.; Martinov M. V.; Vitvitsky V. M.; Banerjee R.; Ataullakhanov F. I. An Allosteric Mechanism for Switching between Parallel Tracks in Mammalian Sulfur Metabolism. PLoS Comput. Biol. 2008, 4, e1000076 10.1371/journal.pcbi.1000076. 18451990 PMC2346559

[ref23] Poirier D. J. Revising Beliefs in Nonidentified Models. Econom. Theor. 1998, 14, 483–509. 10.1017/S0266466698144043.

[ref24] Van Rossum G.; Drake F. L. Python 3 Reference Manual; CreateSpace: Scotts Valley, CA, 2009.

[ref25] Kacser H.; Burns J. A. The control of flux. Symp. Soc. Exp. Biol. 1973, 27, 65–104. 4148886

[ref26] Noor E.; Flamholz A.; Liebermeister W.; Bar-Even A.; Milo R. A note on the kinetics of enzyme action: A decomposition that highlights thermodynamic effects. FEBS Lett. 2013, 587, 2772–2777. 10.1016/j.febslet.2013.07.028. 23892083

[ref27] Liebermeister W.; Uhlendorf J.; Klipp E. Modular rate laws for enzymatic reactions: thermodynamics, elasticities and implementation. Bioinformatics 2010, 26, 1528–1534. 10.1093/bioinformatics/btq141. 20385728

[ref28] Monod J.; Wyman J.; Changeux J. P. On the nature of allosteric transitions: a plausible model. J. Mol. Biol. 1965, 12, 88–118. 10.1016/S0022-2836(65)80285-6. 14343300

[ref29] Changeux J.-P. 50 years of allosteric interactions: the twists and turns of the models. Nat. Rev. Mol. Cell Biol. 2013, 14, 819–829. 10.1038/nrm3695. 24150612

[ref30] Popova S. V.; Sel’kov E. E. Generalization of the model by monod, wyman and changeux for the case of a reversible monosubstrate reaction. FEBS Lett. 1975, 53, 269–273. 10.1016/0014-5793(75)80034-2. 1137953

[ref31] Popova S. V.; Sel’kov E. E. [Description of the kinetics of the two substrate reactions S1+ S2 goes to and comes from S3+S4 by a generalized Monod, Wyman, Changeux model]. Mol. Biol. 1979, 13, 129–139. 156878

[ref32] Matos M. R. A.; Saa P. A.; Cowie N.; Volkova S.; de Leeuw M.; Nielsen L. K. GRASP: A Computational Platform for Building Kinetic Models of Cellular Metabolism. Bioinform. Adv. 2022, 2, vbac066 10.1093/bioadv/vbac066. 36699366 PMC9710608

[ref33] Vehtari A.; Simpson D.; Gelman A.; Yao Y.; Gabry J. Pareto Smoothed Importance Sampling. arXiv 2022, 1507.02646 [stat.CO] 10.48550/arXiv.1507.02646.

[ref34] Dai Z.; Locasale J. W. Understanding Metabolism with Flux Analysis: From Theory to Application. Metab. Eng. 2017, 43, 94–102. 10.1016/j.ymben.2016.09.005. 27667771 PMC5362364

[ref35] GitHub. Click Developers, Click: Python Composable Command Line Interface Toolkit, Pallets, 2022; https://pypi.org/project/click/.

[ref36] Pearson W. Toml: Python Library for Tom’s Obvious, Minimal Language, 2020. https://pypi.org/project/toml/.

[ref37] Pydantic Developers; Pydantic. 2022; https://pypi.org/project/pydantic/.

[ref38] Carpenter B.; Gelman A.; Hoffman M. D.; Lee D.; Goodrich B.; Betancourt M.; Brubaker M.; Guo J.; Li P.; Riddell A. Stan: A Probabilistic Programming Language. J. Stat. Software 2017, 76, 1–32. 10.18637/jss.v076.i01. PMC9788645 36568334

[ref39] GitHub. Stan Development Team, CmdStanPy, 2022. https://github.com/stan-dev/cmdstanpy.

[ref40] Betancourt M. A Conceptual Introduction to Hamiltonian Monte Carlo. arXiv 2018, 1701.02434v2 [stat.ME] 10.48550/arXiv.1701.02434.

[ref41] Kumar R.; Carroll C.; Hartikainen A.; Martin O. ArviZ a Unified Library for Exploratory Analysis of Bayesian Models in Python. J. Open Source Softw. 2019, 4, 1143 10.21105/joss.01143.

[ref42] Timonen J.; Siccha N.; Bales B.; Lähdesmäki H.; Vehtari A. An Importance Sampling Approach for Reliable and Efficient Inference in Bayesian Ordinary Differential Equation Models. arXiv 2022, 2205.09059 [stat.CO] 10.48550/arXiv.2205.09059.

[ref43] Serban R.; Hindmarsh A. C. CVODES: The Sensitivity-Enabled ODE Solver in SUNDIALS Volume 6: 5th International Conference on Multibody Systems, Nonlinear Dynamics, and Control, Parts A, B, and C; ASME: Long Beach, California, USA, 2005; pp 257–269.

[ref44] Margossian C. Computing Steady States with Stan’s Nonlinear Algebraic Solver; Stan Conference, 2018.

