# Supplementary material for: Bayesian Regression Facilitates Quantitative Modeling of Cell Metabolism
Source: ACS Synth Biol. 2024 Apr 5;13(4):1205–14. doi: 10.1021/acssynbio.3c00662 (PMC11036490; doi:10.1021/acssynbio.3c00662)
Supplement: Supplementary file 1 — sb3c00662_si_001.pdf [file sb3c00662_si_001.pdf]

Supporting information - Bayesian regression facilitates quantitative modelling of cell metabolism

Teddy Groves,[*,†] Nicholas Luke Cowie,[†] and Lars Keld Nielsen[‡]

†*The Novo Nordisk Foundation Center for Biosustainability, DTU, Kongens Lyngby 2800, Denmark*

‡*Australian Institute for Bioengineering and Nanotechnology (AIBN), The University of Queensland, St Lucia 4067, Australia*

E-mail: tedgro@biosustain.dtu.dk

This document provides information in support of our article "Bayesian regression facilitates quantitative modelling of cell metabolism".

The results of all reported Maud runs can be found at https://github.com/biosustain/Methionine_model/blob/main/results.

1 Maud's input format

Maud inputs are structured directories, somewhat inspired by the PEtab format (1). A Maud input directory must contain a toml (2) file called `config.toml` which gives the input a name, configures how Maud will be run and tells Maud where to find the other files, allowing these to have custom names. It must also include a file containing a kinetic model definition, a file specifying information about parameters and a file with information experiments. The required structure of these files is documented at https://maud-metabolic-models.readthedocs.io/en/latest/inputting.html. The input is validated against a Pydantic (3) data model.

We chose to implement a custom input format despite the existence of standard formats in similar areas, including SBML (4) and PEtab (1). This choice was partly motivated by the need to ensure flexibility as Maud was developed, but there are also features of SBML and PEtab that make them structurally unsuitable in this context. Our requirements for an input format included that it be mathematics-free, so that all mathematical details are encapsulated in source code, and that it has a detailed, verifiable structure. These requirements made toml more attractive than SBML: toml is easier for humans to read and edit and can straightforwardly be validated using tools like Pydantic. Further, an SBML representation of our desired input would not contain differential equations. It would therefore not be interoperable with most SBML targeting software, which typically assumes that differential equations are available and does not know about Maud's structure.

2 Maud's kinetic model

2.1 Parameters

Table 1 shows all of Maud's unknown parameters along with their dimensions

Note that Maud's metabolic model includes some quantities that are not treated as parameters in its statistical model, including temperatures, compartment volumes and the formation energy of water. Maud treats these quantities as if they were known precisely: they can be configured by the user or default values can be used. Although in practice there can be considerable uncertainty regarding these quantities, we chose to disregard this uncertainty in the interest of simplicity.

Table 1: Table S1 – Parameters of Maud's statistical model

Parameter	Modelled quantity	Dimensions
$\Delta_f G$	Formation energy	metabolites
k_M	Michaelis Menten constants	Substrates of all enzyme/reactions and products of reversible enzyme/reactions
k_I	Inhibition constants	Inhibiting metabolite/compartments of enzyme/reactions exhibiting competitive inhibition
k_{cat}	Rate constants	Enzyme/reactions
L_0	Transfer constants	Allosteric interactions
e_T	T dissociation constants	Modifying metabolites of allosteric inhibitions

Parameter	Modelled quantity	Dimensions
e_R	R dissociation constants	Modifying metabolites of allosteric activations
$k_{cat\,pme}$	Rate constants of phosphorylation modifying enzymes	Phosphorylation modifying enzymes
v_{drain}	Drain fluxes	Drains, experiments
$Enzyme$	Enzyme concentrations	Enzymes, experiments
$C_{unbalanced}$	Unbalanced metabolite/compartment concentrations	Unbalanced metabolite/compartments, experiments
C_{pme}	Phosphorylation modifying enzyme concentrations	Phosphorylation modifying enzymes, experiments
ψ	Membrane potentials	Experiments

Solving the steady state problem for a given set of parameters in an experiment yields a vector $C_{balanced}$ of balanced metabolite concentrations. These are combined with the balanced metabolite concentrations $C_{unbalanced}$ to produce a vector C_{mic} with a concentration for each metabolite/compartment combination.

$\Delta_f G$ parameters can optionally be fixed; this can be useful for computational purposes, as for example to avoid estimating the formation energy of a metabolite about which there is no available information due to it only participating in irreversible reactions.

2.2 Rate equations

As discussed in the main text, Maud's kinetic model decomposes into factors contributing to the flux in a metabolic network in an experiment as shown in equation (1). For succinct-

ness, and since Maud's model assumes that there are no interactions between experiments, we omit any notation referring to experiments below. We also omit any reference to the network's drain reactions: these are modelled as being exactly determined by the values of the parameter vector v_{drain}.

$$F(C;\theta) = Enzyme \cdot k_{cat} \cdot Reversibility \cdot Saturation \cdot Allostery \tag{1}$$

The term *Enzyme* in equation (1) is a vector of non-negative real numbers representing the concentration of the enzyme catalysing each reaction.

The term k_{cat} in equation (1) is a vector of non-negative real numbers representing the amount of flux carried per unit of saturated enzyme.

The term *Reversibility* in equation (1) is a vector of real numbers capturing the impact of thermodynamic effects on the reaction's flux, as shown in equation (2).

$$\begin{aligned} Reversibility &= 1 - \exp(\frac{\Delta_r G + RT \cdot S^T \ln(C_{mic})}{RT}) \\ \Delta_r G &= S^T \Delta_f G + nF\psi \end{aligned} \tag{2}$$

The terms in (2) have the following meanings:

- T is the temperature in Kelvin (a number),
- R is the gas constant (a number),
- $\Delta_r G$ is a vector representing the Gibbs free energy change of each reaction in standard conditions,
- $\Delta_f G$ is a vector representing the standard condition Gibbs free energy change of each metabolite's formation reaction, or in other words each metabolite's 'formation energy'.
- n is a vector representing the number of charges transported by each reaction.

- F is the Faraday constant (a number)
- ψ is a vector representing each reaction's membrane potential (these numbers only matter for reactions that transport non-zero charge)

Note that, for reactions with zero transported charge, the thermodynamic effect on each reaction is derived from metabolite formation energies. This formulation is helpful because, provided that all reactions' rates are calculated from the same formation energies, they are guaranteed to be thermodynamically consistent.

The term n accounts for both the charge and the directionality. For instance, a reaction that exports 2 protons to the extracellular space in the forward direction would have -2 charge. If a negatively charged molecule like acetate is exported in the forward direction, n would be 1.

Note that this way of modelling the effect of transported charge does not take into account that the concentration gradient used by the transport is that of the dissociated molecules. Thus, this expression is only correct for ions whose concentration can be expressed in the model only in the charged form; e.g., protons, K^+, Na^+, Cl^-, etc.

The term $Saturation$ in equation (1) is a vector of non-negative real numbers representing, for each reaction, the fraction of enzyme that is saturated, i.e. bound to one of the reaction's substrates. To describe saturation we use equation (3), which is taken from Liebermeister et al. (*5*) and Noor et al. (*6*). Additionally, this term captures competitive inhibition: as competitive inhibitor concentration increases, the saturation denominator increases, effectively decreasing the saturation of the substrate on the total enzyme pool. Conversely, as the substrate concentration increases this term approaches 1.

$$
\begin{aligned}
Saturation_r &= a \cdot \text{free enzyme ratio} && (3)\\
a &= \prod_{\text{s substrate}} \frac{C^s_{mic}}{k^{rs}_M}\\
\text{free enzyme ratio} &= \begin{cases}
\displaystyle\prod_{\text{s sustrate}} (1 + \frac{C^s_{mic}}{k^{rs}_M})^{S_s r} + \sum_{\text{c inhibitor}} \frac{C^c_{mic}}{k^{rc}_I} & r \text{ irreversible}\\
\displaystyle -1 + \prod_{\text{s sustrate}} (1 + \frac{C^s_{mic}}{k^{rs}_M})^{S_s r} + \prod_{\text{p product}} (1 + \frac{C^p_{mic}}{k^{rp}_M})^{S_p r} + \sum_{\text{c inhibitor}} \frac{C^c_{mic}}{k^{rc}_I} & r \text{ reversible}
\end{cases}
\end{aligned}
$$

The term *Allostery* in equation (1) is a vector of non-negative numbers describing the effect of allosteric regulation on each reaction. Allosteric regulation happens when binding to a certain molecule changes an enzyme's shape in a way that changes its catalytic behaviour. We use equation (4) to describe this phenomenon, following the generalised MWC approach described in Monod et al. (*7*), Changeux (*8*), Popova and Sel'kov (*9*) and Popova and Sel'kov (*10*).

$$
\begin{aligned}
Allostery_r &= \frac{1}{1 + L^r_0 \cdot (\text{free enzyme ratio}_r \cdot \frac{Qtense}{Qrelaxed})^{subunits}} && (4)\\
Qtense &= 1 + \sum_{\text{i inhibitor}} \frac{C^i_{mic}}{e^{ri}_T}\\
Qrelaxed &= 1 + \sum_{\text{a activator}} \frac{C^a_{mic}}{e^{ra}_R}
\end{aligned}
$$

The parameter L_0 in equation (1) is called the transfer constant, and the parameter vectors e_T and e_R are called tense and relaxed dissociation constants respectively.

Finally, the term *Phosphorylation* in equation (1) captures the important effect whereby enzyme activity is altered due to a coupled process of phosphorylation and dephosphorylation. This description achieves a similar behaviour to the MWC formalism for describing allosteric regulation, but using the rates of phosphorylation and dephosphorylation rather than concentrations of metabolites.

$$Phosphorylation_r = (\frac{\alpha}{\alpha + \beta})^{subunits} \tag{5}$$
$$\alpha = \sum_{\text{p phosphoylator}} k^p_{cat\,pme} \cdot C^p_{pme}$$
$$\beta = \sum_{\text{d dephosphoylator}} k^d_{cat\,pme} \cdot C^d_{pme}$$

3 Methionine case study

3.1 Dataset generation

Starting with the model in Saa and Nielsen (*11*), we extracted values for enzyme concentrations, boundary conditions and fluxes. We used these values to generate MCMC samples using Maud using the priors specified in section Section 3.2. When this was finished, we selected one sample with relatively high log probability to use as a ground truth in our case study. These parameter values are shown below in table Table 2. We manually inspected the parameter values to screen for any obviously implausible values; we did not find any of these.

3.2 Prior distributions compared with true parameter values

Table 2 shows the prior distributions we used for independent parameters. The first two columns show the 1% and 99% quantiles of each marginal prior distribution. True parameter

value are shown in column three, and the last column shows the z-score on log scale of the true parameter value according the marginal prior distribution. As can be seen from the table, there are 7 parameters for which the true value is outside the 1%-99% range.

Table 2: Table S2 – Parameter specification, marginal prior distributions and true parameter values used in our case study.

parameter name	1% prior quantile	99% prior quantile	true value	prior Z-score of true value
$e_R^{CBS1,ametc}$	3.430e-06	0.002480	9.3e-05	0.004
$e_R^{GNMT1,ametc}$	3.000e-05	0.002000	2.000e-05	-2.787
$e_R^{MAT3,ametc}$	1.000e-04	0.001000	3.170e-04	0.003
$e_R^{MAT3,met-Lc}$	4.500e-04	0.000800	6.000e-04	0.000
$e_R^{MTHFR1,ahcysc}$	1.120e-07	0.000081	2.000e-06	-0.101
$e_T^{GNMT1,mlthfc}$	1.120e-05	0.008050	2.290e-04	-0.136
$e_T^{MTHFR1,ametc}$	1.120e-07	0.000081	1.500e-05	0.549306
k_{cat}^{AHC1}	1.200e+02	400.000000	2.340e+02	0.179861
k_{cat}^{BHMT1}	6.000e+00	35.000000	1.380e+01	-0.135
k_{cat}^{CBS1}	1.000e+01	188.000000	7.020e+00	-2.887
k_{cat}^{GNMT1}	7.000e-01	60.000000	1.050e+01	0.352083
k_{cat}^{MAT1}	8.200e-02	59.100000	7.900e+00	0.44375
k_{cat}^{MAT3}	5.890e-01	424.000000	1.990e+01	0.080556
$k_{cat}^{METH-Gen}$	4.840e-01	349.000000	1.160e+00	-1.209

parameter name	1% prior quantile	99% prior quantile	true value	prior Z-score of true value
$k_{cat}^{MS1kcatMS1}$	1.000e+00	3.300000	1.770e+00	-0.091
k_{cat}^{MTHFR1}	1.300e+00	4.200000	3.170e+00	0.183333
k_{cat}^{PROT1}	1.590e-01	0.222000	2.650e-01	0.41875
$k_{I}^{GNMT1,ahcysc}$	2.000e-06	0.001400	5.300e-05	0.010
$k_{I}^{MAT1,ametc}$	3.000e-04	0.000400	3.470e-04	0.014
$k_{I}^{METH-Gen,ahcysc}$	1.000e-06	0.000030	6.000e-06	0.021
$k_{M}^{AHC1,ahcysc}$	5.220e-05	0.037600	2.320e-05	-2.050
$k_{M}^{AHC1,adnc}$	1.670e-07	0.000120	5.660e-06	0.081944
$k_{M}^{AHC1,hcys-Lc}$	1.580e-07	0.000114	1.060e-05	0.318056
$k_{M}^{BHMT1,hcys-Lc}$	1.200e-05	0.000032	1.980e-05	0.049
$k_{M}^{BHMT1,glybc}$	4.720e-05	0.034000	8.460e-03	0.659028
$k_{M}^{CBS1,hcys-Lc}$	1.000e-06	0.000025	4.240e-05	3.090
$k_{M}^{CBS1,ser-Lc}$	2.000e-06	0.000004	2.830e-06	0.004

parameter name	1% prior quantile	99% prior quantile	true value	prior Z-score of true value
$k_M^{GNMT1,ametc}$	1.300e-05	0.009400	5.200e-04	0.1375
$k_M^{GNMT1,ahcysc}$	4.100e-07	0.000295	1.100e-05	0.000
$k_M^{GNMT1,glyc}$	5.480e-05	0.039500	2.540e-03	0.189583
$k_M^{GNMT1,sarcsc}$	3.730e-09	0.000003	1.000e-07	0.000
$k_M^{MAT1,met-Lc}$	1.400e-05	0.000720	1.070e-04	0.074
$k_M^{MAT1,atpc}$	5.270e-05	0.038000	2.030e-03	0.125694
$k_M^{MAT3,met-Lc}$	4.470e-05	0.032200	1.130e-03	-0.029
$k_M^{MAT3,atpc}$	5.270e-05	0.038000	2.370e-03	0.179167
$k_M^{METH-Gen,ametc}$	7.000e-06	0.000013	9.370e-06	-0.135
$k_M^{MS1,5mthfc}$	3.320e-06	0.002390	6.940e-05	-0.124
$k_M^{MS1,hcys-Lc}$	1.000e-06	0.000003	1.710e-06	-0.054
$k_M^{MTHFR1,mlthfc}$	7.500e-05	0.000088	8.080e-05	-0.158

parameter name	1% prior quantile	99% prior quantile	true value	prior Z-score of true value
$k_M^{MTHFR1,nadphc}$	1.600e-05	0.000028	2.090e-05	-0.105
$k_M^{PROT1,met-Lc}$	4.500e-05	0.000085	4.390e-05	-2.507
L_0^{CBS1}	3.730e-02	26.800000	1.030e+00	0.017
L_0^{GNMT1}	3.730e-02	26.800000	1.310e+02	0.3875
L_0^{MAT3}	3.730e-03	2.680000	1.080e-01	0.037
L_0^{MTHFR1}	1.120e-01	80.500000	3.920e-01	-1.018

$\Delta_f G$ parameters for most metabolites were fixed; those that were modelled as unknown had a multivariate normal prior distribution derived from eQuilibrator (*12*).

The values for $\Delta_f G$ parameters, as well as all other model parameters, can be found by inspecting the file `priors.toml` which is online at https://github.com/biosustain/Methionine_model/blob/main/data/methionine/priors.toml.

3.3 Computation

We conducted adaptive Hamiltonian Monte Carlo sampling for the full and missing-data datasets. For the full dataset we obtained 1000 post-warmup samples each from 4 independent Markov chains after 1000 warm-up samples and "hot-starting" with a mass metric output by a previous model run.

For the missing-data dataset 250 post-warmup samples were taken from 4 indpendent Markov chains after 100 warmup samples. The sampling was initialised using the mass matric from

the complete measurement dataset and the warmup consisted of step size adaption for 100 samples. The resulting posterior distribution had an $\hat{R} = 1.01$ for the log-probability and did not exhibit post-warmup divergences that were not a result of differential equation errors.

4 Laplace approximation case study

To compare MCMC sampling with Laplace approximation we used a different model with fewer parameters and state variables. This model was chosen because we were not able to generate results for our methionine model using Laplace approximation. The simpler case still serves to illustrate the general issues with approximating the posterior distributions of Bayesian kinetic models using the Laplace method, and that the associated numerical instability is another reason to prefer other methods where possible.

The full Maud input folders used for our Laplace approximation case study can be found at https://github.com/biosustain/Methionine_model/tree/main/data/example_ode and https://github.com/biosustain/Methionine_model/tree/main/data/example_ode_laplace.

To generate Laplace samples we used Maud's Laplace mode.

5 Multimodal posterior distributions

While we have not yet observed this in practice, we expect that Maud is capable of accurately sampling mildly multi-modal posterior distributions, i.e. those for which regions of parameter space with high probability density are not very sharply separated. This is because this ability depends primarily on the underlying inference algorithm, and adaptive Hamiltonian Monte Carlo is known to be able to sample many such posterior distributions. See, for example, Mangiola et al. (*13*), which reports the successful use of adaptive Hamiltonian Monte Carlo to sample posterior distributions involving mixtures of Gaussian distributions, which are typically multi-modal.

Moreover, in cases where it fails to sample a multi-modal posterior distribution adaptive Hamiltonian Monte Carlo typically exhibits poor mixing and divergent transitions, which Maud is set up to detect automatically, making this case easy for users to diagnose.

Nonetheless, it is also well known that strongly multi-modal posterior distributions pose a problem for adaptive Hamiltonian Monte Carlo, as well as other MCMC algorithms. See (*14*, §3.2) for a general discussion of this issue. Mangoubi et al. (*15*) explores the related question of the relative performance of Hamiltonian Monte Carlo vs random walk Metropolis Hastings, finding that, for certain cases where the target distribution exhibits highly disconnected and similarly shaped modes, the two algorithms have similar performance.

It is therefore important to pay careful attention to MCMC diagnostics and monitor developments in computational statistics that might expand the range of posterior distributions that can practically be sampled.

Zamora-Sillero et al. (*16*) introduces the method HYPERSPACE, which is designed to identify disconnected regions of a biochemical parameter space and characterise them by estimating their dimensions. HYPERSPACE does not perform parameter inference and is therefore not directly comparable with algorithms like adaptive Hamiltonian Monte Carlo as used in Maud, but could plausibly be adapted into such an algorithm, for example by supplementation with importance sampling (see Vehtari et al. (*17*)). However, any such algorithm would be limited by HYPERSPACE's dependency on a random walk Metropolis Hastings algorithm. This kind of algorithm's performance is known to scale poorly compared with Hamiltonian Monte Carlo algorithms as the number of parameters increases: see Mangoubi et al. (*15*). Since Maud aims to fit models with hundreds of parameters, this means that it is unlikely that HYPERSPACE can directly be used to improve Maud's efficiency.

6 References

1. Schmiester, L. et al. (2021) PEtab—Interoperable Specification of Parameter Estimation Problems in Systems Biology. *PLOS Computational Biology 17*, 1–10.

2. Preston-Werner, Tom and Gedam, Pradyun TOML Specification 1.0.0-Rc.1. 2020; https://toml.io/en/v1.0.0-rc.1/.

3. Pydantic developers Pydantic. 2022; https://pypi.org/project/pydantic/.

4. Keating, S. M. et al. (2020) SBML Level 3: An Extensible Format for the Exchange and Reuse of Biological Models. *Molecular Systems Biology 16*, e9110.

5. Liebermeister, W., Uhlendorf, J., and Klipp, E. (2010) Modular rate laws for enzymatic reactions: thermodynamics, elasticities and implementation. *Bioinformatics 26*, 1528–1534.

6. Noor, E., Flamholz, A., Liebermeister, W., Bar-Even, A., and Milo, R. (2013) A note on the kinetics of enzyme action: A decomposition that highlights thermodynamic effects. *FEBS Letters 587*, 2772–2777.

7. Monod, J., Wyman, J., and Changeux, J. P. (1965) On the nature of allosteric transitions: a plausible model. *Journal of Molecular Biology 12*, 88–118.

8. Changeux, J.-P. (2013) 50 years of allosteric interactions: the twists and turns of the models. *Nature Reviews. Molecular Cell Biology 14*, 819–829.

9. Popova, S. V., and Sel'kov, E. E. (1975) Generalization of the model by Monod, Wyman and Changeux for the case of a reversible monosubtrate reaction $S\overleftrightarrow{(R,T)}P$. *FEBS Letters 53*, 269–273.

10. Popova, S. V., and Sel'kov, E. E. (1979) [Description of the kinetics of the two substrate reactions S1+ S2 goes to and comes from S3+S4 by a generalized Monod, Wyman, Changeux model]. *Molekuliarnaia Biologiia 13*, 129–139.

11. Saa, P. A., and Nielsen, L. K. (2016) Construction of feasible and accurate kinetic models of metabolism: A Bayesian approach. *Scientific Reports 6*, 29635.

12. Beber, M. E., Gollub, M. G., Mozaffari, D., Shebek, K. M., and Noor, E. eQuilibrator 3.0 – a Platform for the Estimation of Thermodynamic Constants. 2021; http://arxiv.org/abs/2103.00621.

13. Mangiola, S., Roth-Schulze, A. J., Trussart, M., Zozaya-Valdés, E., Ma, M., Gao, Z., Rubin, A. F., Speed, T. P., Shim, H., and Papenfuss, A. T. (2023) Sccomp: Robust Differential Composition and Variability Analysis for Single-Cell Data. *Proceedings of the National Academy of Sciences 120*, e2203828120.

14. Betancourt, Michael *Markov Chain Monte Carlo*; 2020.

15. Mangoubi, O., Pillai, N. S., and Smith, A. Does Hamiltonian Monte Carlo Mix Faster than a Random Walk on Multimodal Densities? 2018; http://arxiv.org/abs/1808.03230.

16. Zamora-Sillero, E., Hafner, M., Ibig, A., Stelling, J., and Wagner, A. (2011) Efficient Characterization of High-Dimensional Parameter Spaces for Systems Biology. *BMC Systems Biology 5*, 142.

17. Vehtari, A., Simpson, D., Gelman, A., Yao, Y., and Gabry, J. Pareto Smoothed Importance Sampling. 2022; http://arxiv.org/abs/1507.02646.
